# Lower amyloid and small vessel disease in unimpaired older adults with Down syndrome

**DOI:** 10.1002/alz70856_099396

**Published:** 2025-12-24

**Authors:** Patrick J. Lao, Anna C. Smith, Natalie C. Edwards, Lisi Flores‐Aguilar, Mohamad J. Alshikho, Juyoung Hahm, Dana L Tudorascu, H. Diana Rosas, Sigan L Hartley, Michael A. Yassa, Benjamin L Handen, Mark Mapstone, Bradley T Christian, Jose Gutierrez, Donna M. Wilcock, Elizabeth Head, Adam Brickman

**Affiliations:** ^1^ Columbia University Irving Medical Center, New York, NY, USA; ^2^ Taub Institute for Research on Alzheimer's Disease and the Aging Brain, New York, NY, USA; ^3^ University of California, Irvine, Irvine, CA, USA; ^4^ University of Pittsburgh, Pittsburgh, PA, USA; ^5^ Athinoula A. Martinos Center for Biomedical Imaging, Massachusetts General Hospital, Harvard Medical School, Charlestown, MA, USA; ^6^ Waisman Center, University of Wisconsin‐Madison, Madison, WI, USA; ^7^ Indiana University School of Medicine, Stark Neurosciences Research Institute, Department of Neurology, Indianapolis, IN, USA

## Abstract

**Background:**

The revised Alzheimer's Association Research Framework presumes that all adults with Down syndrome (DS) are on the Alzheimer's disease (AD) continuum, developing AD at a median age of 54 years old after lifelong overproduction of amyloid precursor protein. However, some older adults with DS “escape” AD biomarker development or cognitive impairment, even in the context of genetically determined AD risk. We explored whether older adults with DS without a clinical diagnosis or with lower‐than‐expected amyloid differed in their tau and cerebrovascular disease burden.

**Method:**

Adults with DS (*n* = 259; 45±10 yrs, 43% women, 75% Cognitively‐Stable/12% MCI‐DS/13%AD‐DS) from the Alzheimer's Biomarker Consortium‐Down syndrome (ABC‐DS) underwent vascular MRI (white matter hyperintensity (WMH), enlarged perivascular space (PVS), infarcts, microbleeds), amyloid PET, and tau PET. Adults with DS who were Cognitively‐Stable above the age of 54 years old (i.e., greater‐than‐expected cognition for their age) were categorized separately from all others. All biomarkers were fit against left‐null piecewise regression models with age, adjusting for site; the lower 5^th^ percentile of age‐trajectory residuals (i.e., less‐than‐expected for their age) for amyloid were categorized separately from the upper 95^th^ percentile. Participants with better‐than‐expected cognition and amyloid were compared in terms of demographic characteristics as well as tau and cerebrovascular residuals.

**Result:**

Older Cognitively‐Stable adults were older by definition (15 [10, 19], *p* =  2E‐9), had lower age‐residuals for amyloid (‐27 [‐44, ‐9], *p* = 0.003), and temporal (‐0.3 [‐0.6, ‐0.01], *p* = 0.04), parietal (‐1.4 [‐2.5, ‐0.2], *p* = 0.02), and occipital WMH (‐0.8 [‐1.5, ‐0.06], *p* = 0.03). Individuals in the 5^th^ percentile of age‐residuals for amyloid were older (11 [5, 17], *p* = 0.0003), had lower age‐residuals for tau in early Braak tau (‐0.2 [‐0.4, ‐0.1], *p* = 0.0002), middle Braak (‐0.3 [‐0.4, ‐0.1], *p* = 0.0002), late Braak (‐0.2 [‐0.4, ‐0.06], *p* = 0.007), as well as deep microbleeds (‐1.0 [‐0.2, ‐0.04], *p* = 0.002).

**Conclusion:**

Older adults with DS who remain cognitively‐stable had lower amyloid and posterior WMH, while those that maintain low amyloid at older ages had lower tau and microbleeds. Future work will investigate protective factors that may contribute to biomarker and clinical variability, even in the context of genetically determined AD risk.

